# Take Only Photographs, Leave Only Footprints: Novel Applications of Non-Invasive Survey Methods for Rapid Detection of Small, Arboreal Animals

**DOI:** 10.1371/journal.pone.0146142

**Published:** 2016-01-20

**Authors:** Cheryl A. Mills, Brendan J. Godley, David J. Hodgson

**Affiliations:** 1 Centre for Ecology and Conservation, College of Life and Environmental Sciences, University of Exeter, Cornwall Campus, Penryn, Cornwall, United Kingdom; 2 School of Life Sciences, Keele University, Keele, Staffordshire, United Kingdom; University of Tasmania, AUSTRALIA

## Abstract

The development of appropriate wildlife survey techniques is essential to promote effective and efficient monitoring of species of conservation concern. Here, we demonstrate the utility of two rapid-assessment, non-invasive methods to detect the presence of elusive, small, arboreal animals. We use the hazel dormouse, *Muscardinus avellanarius*, a rodent of conservation concern, as our focal species. Prevailing hazel dormouse survey methods are prolonged (often taking months to years to detect dormice), dependent on season and habitat, and/or have low detection rates. Alternatives would be of great use to ecologists who undertake dormouse surveys, especially those assessing the need for mitigation measures, as legally required for building development projects. Camera traps and footprint tracking are well-established tools for monitoring elusive large terrestrial mammals, but are rarely used for small species such as rodents, or in arboreal habitats. In trials of these adapted methods, hazel dormice visited bait stations and were successfully detected by both camera traps and tracking equipment at each of two woodland study sites, within days to weeks of installation. Camera trap images and footprints were of adequate quality to allow discrimination between two sympatric small mammal species (hazel dormouse and wood mouse, *Apodemus sylvaticus*). We discuss the relative merits of these methods with respect to research aims, funds, time available and habitat.

## Introduction

Biological surveys and monitoring programs are essential for acquiring knowledge of natural systems. Objectives include identifying trends in population size and range, habitat modelling, habitat use studies, evaluating ecological management approaches and biodiversity assessment [1, 2]. Many research questions can be addressed through simple presence surveys, avoiding the need for complex abundance estimates, which are substantially more costly in time and effort [3].

Technologically advanced monitoring tools, such as remote camera traps, are increasingly being used, as they become more accessible and affordable [4, 5]. The advantages of camera trapping include non-invasiveness, low surveyor time required and the provision of relatively unambiguous, permanent records, for species that are difficult to observe. However, equipment failure, user-error and initial expense can be problematic [6–8]. Despite the increased use of camera traps, they are still not meeting their potential in ecological research [4, 6]. We conducted a search of the ISI Web of Knowledge database, for the term “camera trap” (in the subject areas: Environmental Science, Zoology and Biodiversity and Conservation) and selected those concerning at least one terrestrial mammal species. Of the 367 entries, 91% of the studies focussed on medium/large species only, 6% on multi-species surveys and just 3% on small mammals (<200g) alone. Whilst smaller vertebrates have a lower capture probability [8–10] camera trapping has been shown to be feasible for small mammal surveying [11, 12] and therefore warrants further research and utilisation.

Less technologically-sophisticated methods for collecting information on wildlife presence and activity include surveying sites for animal tracks. To circumvent the difficulty of finding footprints in the environment, animals can be attracted to track-collecting equipment, often using lures or bait (e.g. [13–15]). Tracking stations have been used to survey many terrestrial species including rodents [16], insectivores [17], mustelids [18], lizards [19] and insects [20]. Additionally, tracking tunnels have been adapted for aquatic mammals [21], but to date have rarely been employed in arboreal habitats (but see [22, 23]). These methods are relatively cheap and easy to install, therefore allowing a large survey effort, but require expertise and time for footprint identification. Recent advances in the statistical analysis of footprints for species and even individual identification (e.g. [24, 25]) are providing new, objective and rapid tools for such analysis, greatly increasing the potential of tracking monitoring.

Our goal was to test, and if successful promote, the use of camera trapping and footprint tracking methods for determining the presence of small, arboreal mammals, using the hazel dormouse, *Muscardinus avellanarius* as our focal species. The hazel dormouse is difficult to study, owing to its elusive nature, small size, low population densities and nocturnal, arboreal behaviour [26]. As a European protected species, the impact of development, land-use change or habitat management upon dormice must be assessed and mitigated [27], often with some urgency. However, current dormouse survey techniques are seasonal, habitat dependent and often prolonged [27, 28]. Nest boxes and nest tubes are the established tools for monitoring dormice in the UK, but the lag between their installation within a habitat and uptake by dormice can be measured in months or even years. Their efficacy also varies with habitat conditions, for example they may be used infrequently by dormice if many natural nesting sites are available [28]. A visual record of the animal in a nest box or tube is highly reliable, but is invasive and requires a handling licence in the UK [27]. Hair tube surveys and nest searches are more economical, but have a low detection rate [29]. Searches for evidence of dormouse feeding on hazelnut shells are only suitable at sites with sufficient fruiting hazel trees, and are best carried out in the late summer to early winter [27]. Commercial pressures and contractual obligations, along with time and budget constraints, may result in conflicts between development requirements and Ecological Impact Assessments [30]. There is, therefore, a pressing need for simple, inexpensive and accurate methods for the rapid detection of hazel dormice.

## Materials and Methods

### Study Sites

The investigation was conducted at two Cornwall Wildlife Trust reserves located in the south-west UK, where dormice were known to be present from monthly checks of dedicated nest boxes. Cabilla is a site of ancient mixed woodland with areas of oak and hazel coppice. Red Moor is a reserve of heath and grassland with areas of woodland, including hazel coppice. Based on nest box surveys undertaken by the authors and others as part of the National Dormouse Monitoring Programme, the frequency of nest box use by dormice was significantly higher at Cabilla compared to Red Moor prior to, and during, the survey year, which suggests a greater density of dormice at the former site [31]. Therefore, in order to enhance detection probability during this pilot study, the initial trials were conducted at Cabilla, and further trials then undertaken at Red Moor to test the methodology at a site with assumed lower dormouse density. It should be noted, however, that unmeasured differences in habitat, such as natural nesting site availability, may also account for the variation in the use of nest boxes by dormice between sites, rather than dormouse density.

### Camera traps

Five Scoutguard SG550 (HCO Outdoor Products, Georgia, USA) trail camera traps were used in this study. These are passive infrared heat and motion triggered cameras with an infrared flash, which is less detectable by animals than a white flash, although it should be noted they may still be seen and/or heard by animals [32]. Prior to field trials, the camera traps were piloted in a garden setting to ascertain whether the image quality would be sufficient to detect and discriminate between small mammal species. It was determined that camera traps should be placed approximately 1–1.5 meters from the bait, to produce a clear image large enough to identify small species. At this proximity the infra-red flash over-exposed the image in our camera model and so was covered with opaque tape to reduce flash intensity.

Camera traps were set to take video footage of 20 seconds duration once triggered, with a delay of 1 minute between triggers to conserve memory. Video was chosen over stills as the former allows the capture of many frames of images, increasing the chances of species detection and identification. The trade-off associated with video capture is that camera trap memory cards are filled more rapidly, forcing more regular checks. Small mammal species, specifically the hazel dormouse and wood mouse, *Apodemus sylvaticus*, were identified based on morphological features such as ear size, tail length, head shape and the presence of fur on the tail.

### Tracking cages

**Fig 1:**
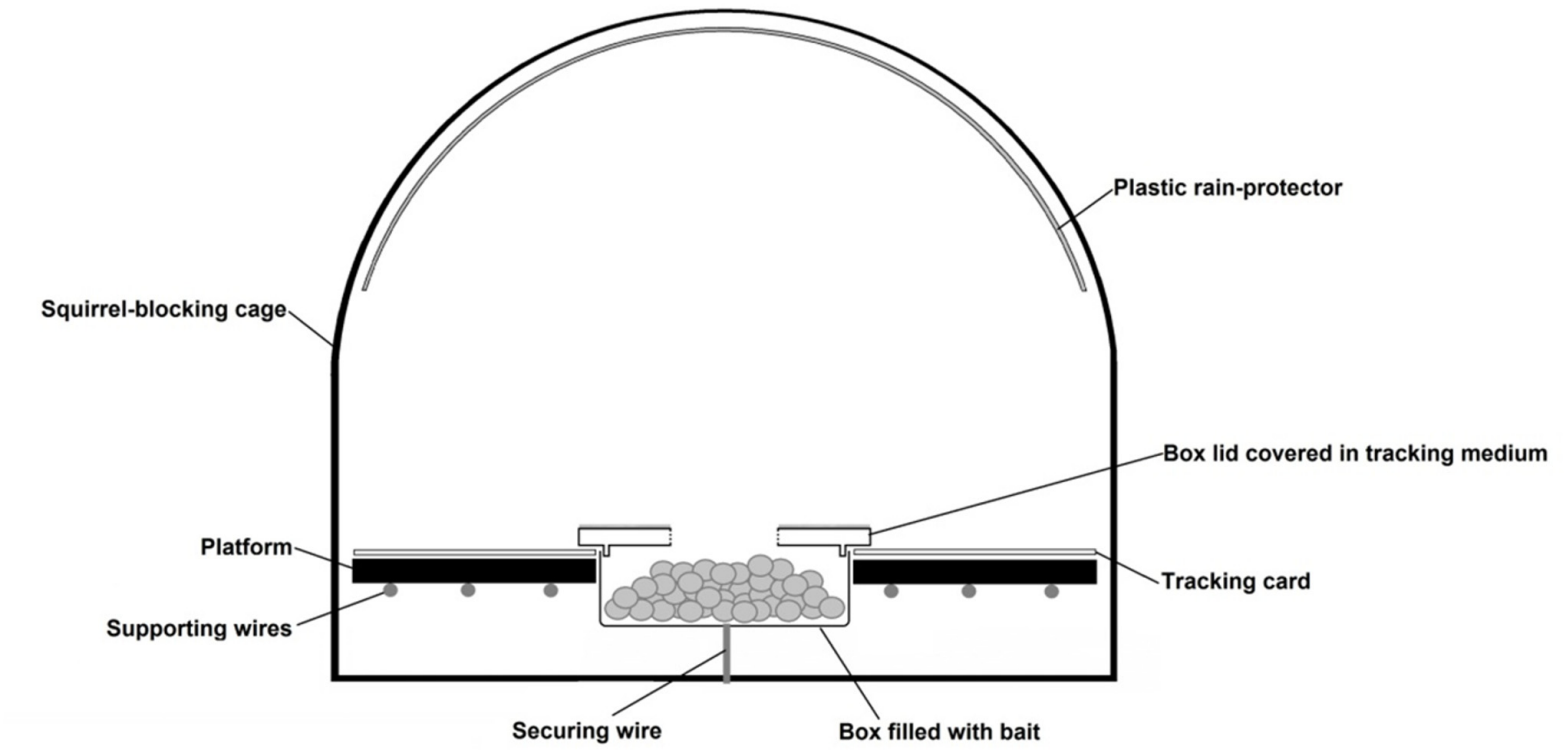
Schematic diagram of a longitudinal section through the footprint tracking cage. The securing wire holds the bait box to the bottom of the cage. A network of supporting wires provides a frame for the platform. A hole in the lid of the bait box provides allow small mammals access to the bait. The tracking card is a circular piece of card with a hole in the middle which fits around the bait box. The inner edge of hole in the tracking card fits underneath the outer rim of the box lid, which helps to hold it in place. The plastic cover protects the tracking equipment from rain damage.

We designed and built five baited tracking cages to collect small animal prints in the tree canopy (Fig 1 and S1 Fig), using adapted 8-inch squirrel blocking cages (Chapelwood, Worcestershire, UK). The cage was required to prevent non-target grey squirrels (*Sciurus carolinensis*) from depleting bait and inundating tracking cards with footprints.

A platform inside the lower half of the cage was constructed by supporting a piece of round rigid corrugated plastic sheeting by a framework of wire. A plastic bait box was fitted tightly into an aperture within the platform. A replaceable square of white card (180gsm) with an aperture that fitted around the plastic box rim was placed on the platform and secured in place by the box lid. The lid of the box was covered in tracking medium, which comprised graphite powder mixed with sunflower oil to a viscous consistency and a hole in the lid allowed small animals to access the bait (sunflower seeds, peanuts and apple pieces) inside. Plastic sheeting was attached to the ceiling of the cage to protect the platform from rain. Two to three drops of honeysuckle oil were applied to a piece of foam sponge and attached to the cage as an additional scent lure. Animals should be attracted to the bait/lure, and those small enough to fit into the cage walk over the tracking medium when accessing the bait, leaving tracks on the card when they depart. Cards are later retrieved, tracks fixed and replaced with new card.

All tracking cards with footprints were scanned by eye to identify clear prints with a minimum of four toe marks visible. These were photographed next to a precision scale and identified, using reference footprints as a comparison (S2 Fig). Dormouse reference footprints were collected from captive animals at Paignton Zoo. Wood mouse and bank vole reference footprints were collected from animals live-trapped during other studies.

### Study Programme

The field testing of baiting, camera traps and tracking equipment occurred in three phases, in order to investigate several questions regarding the survey of small arboreal mammals: 1) Do bait stations attract such species? 2) Are camera trap images sufficiently clear to identify species? 3) Do tracking cages collect tracks of adequate quality to allow discrimination between species? 4) If so, how soon after installation of the bait stations, and how frequently, are small arboreal mammals detected visiting monitoring stations? 5) How do detection rates compare between camera traps and tracking cages?

At both survey sites monitoring stations were distributed within the nest box survey site at a minimum of 60 metres apart. During all phases of the study, monitoring equipment was trialled over a series of consecutive trapping sessions, each comprising, on average, 2.53 trapping nights (range 1–6 nights). This variation in number of trapping nights per trapping session was due to logistics/weather, dictating when the site could be accessed. At the end of each trapping session, bait, lure, footprint cards and camera trap batteries were replenished in preparation for the next trapping session, and camera trap footage and/or tracks were collected.

During phase one, our objectives were to establish whether small, arboreal mammals would be attracted to the lure/bait and investigate the ability of camera traps to provide sufficiently clear images to allow species discrimination. Between the nights of 6^th^ July and 25^th^ July 2010 five monitoring stations with camera traps were installed and set at Cabilla. Each station comprised of a baited tray (a wooden frame with a mesh floor) with honeysuckle lure, hung from tree branches approximately 2.5 meters above ground level and one camera trap aimed at the tray. Camera traps were secured with Python^™^ adjustable locking cables (Masterlock, Neuilly-sur-Seine, France).

In phase two, surveys were conducted on the nights of 5^th^ August to the 2^nd^ September 2010. Once the effectiveness of the bait trays and camera traps had been confirmed in phase one, we swapped the trays for tracking cages at the five monitoring stations. This allowed us to determine if tracking cages could collect clear, identifiable footprints from small, arboreal mammals. Distinct phases one and two were used to ensure the novel tracking equipment did not bias objectives of phase one. The camera traps remained monitoring at the stations, to allow comparison of detection rates between camera traps and footprint cages.

In phase three, from 11^th^ September to 12^th^ October 2010, all five monitoring stations were moved to a second site, Red Moor, for testing, which allowed further concurrent comparisons of camera trapping and tracking cages, at a site where the animals would not have been previously habituated to any of the survey equipment.

### Analysis

Descriptive statistics were used to report survey effort and the results obtained by the camera traps and tracking cages in detecting small arboreal mammal presence during this pilot study. Time from installation to first detection of each species was calculated, to determine how rapidly small arboreal mammals start utilising bait stations and therefore how soon presence may be inferred. This was undertaken using camera trap data, as the video timestamp allowed determination of the exact trapping night animals were recorded, rather than simply the trapping session.

Camera trap and tracking cage detection rates, for the period when both techniques were running simultaneously at each site, were compared by calculating the percentage of trapping sessions which detected each of the small mammal species at the two survey sites. Further analysis was conducted using Cohen’s Kappa statistic, to determine if any observed agreement was due to chance alone [33]. This allowed an assessment of the degree of agreement between the two techniques, following guidelines outlined by Landis and Koch [34] and suggested the rate of detection failure for the two methods. Finally, we tested for a correlation of small mammal presence detection between the techniques. Two Spearman’s rank-order correlation tests were performed in R version 2.15.1 [35] on the frequency of trapping sessions that paired camera traps and tracking cages at the same monitoring stations detected a) dormice and b) wood mice during phases two and three combined.

### Ethics Statement

The study has been approved by the College of Life & Environmental Sciences (Penryn) Ethics Committee at the University of Exeter. Captive hazel dormice were kept by Paignton Zoo as part of a conservation reintroduction scheme, licenced by Natural England and at all times acting within the laws of the UK and abiding by all ethical policies of the British and Irish Association of Zoos and Aquariums, the European Association of Zoos and Aquaria and the World Association of Zoos and Aquaria. Collection of dormouse reference footprints took place during normal husbandry practices, when animals would normally be removed from their enclosures, to ensure no additional disturbance to the animals occurred. No licencing was required from Natural England for the field surveys, as they did not involve any activities that would capture, kill or disturb hazel dormice (a European protected species) or damage their resting places. Cornwall Wildlife Trust, the survey site owners, gave permission to conduct the study at Cabilla and Red Moor. All wild wood mice and voles were live trapped following recommended guidelines [36], footprints were collected in the field and animals immediately released at the capture site.

## Results

### Survey effort

Overall, we carried out 32 trapping sessions with five bait stations, over 81 nights, giving a total of 405 trapping nights. Table 1 provides a summary of survey effort over the three testing phases.

**Table 1:** Summary of survey effort and study phases performed to pilot camera traps and footprint tracking to detect small, arboreal mammals. A trapping session comprised of one or more trapping nights, after which data were collected and equipment replenished and reset. Each phase comprised five survey stations. The total number of stations (n = 5) that detected each species, and the median number of trapping nights to first detection of hazel dormice and wood mice by camera traps after first installation are presented with phase 1 and 2 combined, as monitoring stations remained at the same location during these phases.

Phase	Date	Site	Survey method(s)	Total Number of trapping sessions	Total Number of trapping nights	Average number of nights per trapping session (range)	Total Number of trapping nights per phase	Number of stations (n = 5) that detected each species		Median number of trapping nights to first detection(Interquartile Range)	
								Dormice	Wood mice	Dormice	Wood mice
1	6/07-25/07	Cabilla	Camera traps	10	20	2.0 (1–3)	100	5	5	13 (6–15)	10 (7–24.5)
2	5/08-2/09	Cabilla	Tracking cages & camera traps	10	29	2.90 (1–6)	145				
3	11/09-12/10	Red Moor	Tracking cages & camera traps	12	32	2.67 (2–5)	160	2	4	11.5 (2–21)	4 (3–6)

### Success of baiting and camera traps

**Fig 2:**
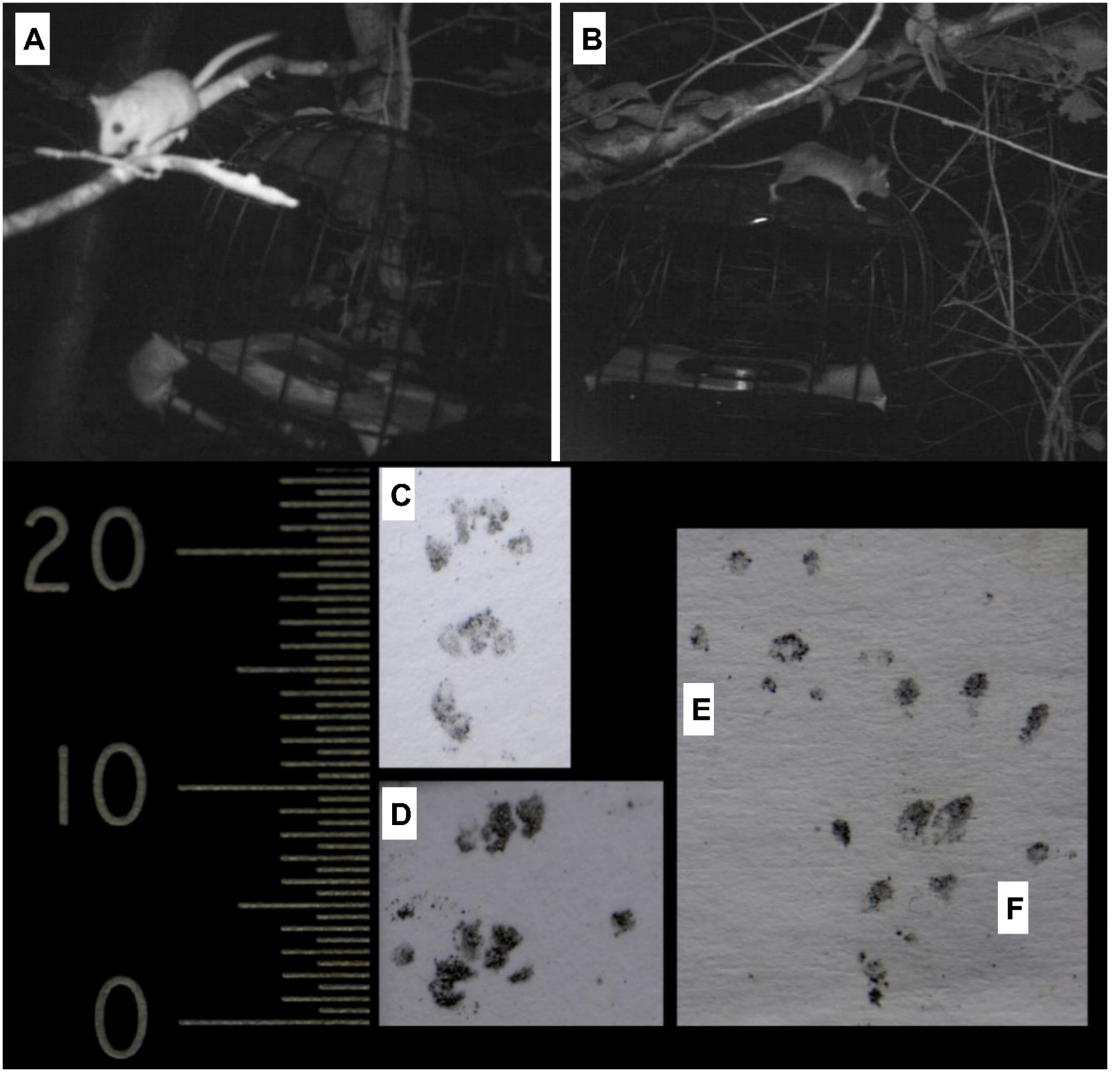
Samples of monitoring results. Frames from camera trap video footage during phase two of study, (therefore includes tracking cages in shots) of: A) two dormice and; B) wood mouse, to demonstrate video quality sufficient to allow species identification. Footprints from wild animals, captured using the footprint tracking cage and subsequently identified as: C) hazel dormouse fore foot and D) dormouse hind foot and E) wood mouse forefoot and F) wood mouse hind foot. All prints are positioned with toes at the top, scale bars represent 0.5mm graduations. Note the distinctive three triangular metacarpal pads found in dormouse prints, which in some prints merge into each other, such as in print C.

We successfully demonstrated arboreal small mammals were attracted to bait and that camera traps captured images sufficiently clear to identify small mammal species (Fig 2a). Over the three study phases 3732 video shots were recorded. Of these, 8.3% captured dormice, and 38.0% wood mice. Conversely, the percentage of shots where no species was identified was 53.7%. It is not possible to ascertain the precise cause for all the negative shots, but they are likely to be due to false triggers, the animal moving out of shot or the image being of too poor quality to allow species identification.

### Time to first detection

We analysed time to first detection at Cabilla (phase one and two combined) and Red Moor (phase three), using camera trap data (Table 1). Out of a possible ten camera stations (five at Cabilla during phases one and two, and five at Red Moor during phase three), seven provided footage of dormice and nine provided footage of wood mice. Across both sites, for those stations that detected each species, the median number of trap nights to first detection was 13 for dormice (interquartile range 6–15) and 8 (interquartile range 4–13) for wood mice.

### Success of tracking cages

It was also successfully demonstrated that tracking cages were able to collect tracks of small mammals, and that they were of adequate quality to allow species identification. Fig 2b displays some foot prints obtained from the tracking cages whilst in the field. The blocking cage also effectively prevented bait disruption by grey squirrels during our study, with only two out of the 305 total trapping sessions during phases two and three being disrupted, due to squirrels being able to open the tracking cage.

Of these 305 total trapping sessions, 65% resulted in tracking cards with at least one print that was sufficiently clear to allow species identification at each bait station. Of the remaining 35% no prints were present; this would be due to either no animals visiting the tracking cage, or failure to collect identifiable prints from animal visitors. Further, whilst there were many overlapping prints, an average of 4 prints per tracking card (SD 3.73, range 1–23 prints) were sufficiently clear to allow an attempt at species identification from a visual scan of each tracking card. These 306 prints were identified by eye. This was achieved by comparing unknown prints to the known reference prints (S2 Fig).

### Camera trap and footprint technique comparison

We compared the detection rate between camera traps and tracking cages, by calculating the percentage of trapping sessions that detected the two species during phases two and three. Note this comparison could not be calculated for phase one as tracking cages were not employed during this phase. For phase two, at Cabilla, the percentage of total trapping sessions (n = 50) that detected dormice and wood mice respectively was 42.0% and 42.0% for camera traps, and 40.0% and 40.0% for tracking cages. In comparison, during phase three at Red Moor the percentage of total trapping sessions (n = 60) that detected dormice and wood mice respectively was 10.0% and 58.3% for camera traps, and 3.33% and 70% for tracking cages.

There was substantial agreement between the two survey methods in detecting both species (Cohen's kappa = 0.68), with 85% of the trapping sessions having an overall agreement. When analysing species separately there was also substantial agreement between techniques for identifying both dormice (Cohen's kappa = 0.61) and wood mice (Cohen's kappa = 0.67). The lower agreement for dormice is probably due to fewer dormice being detected at Red Moor, causing an increased chance that by random both techniques would fail to detect dormice during a trapping session. If we assume that discrepancies were not caused by false-positives and that there were no occasions where both techniques missed small mammal activity, we can conclude that tracking cages failed to detect visiting small mammals in 15% of trapping sessions, and camera traps in 16% of trapping sessions.

**Fig 3:**
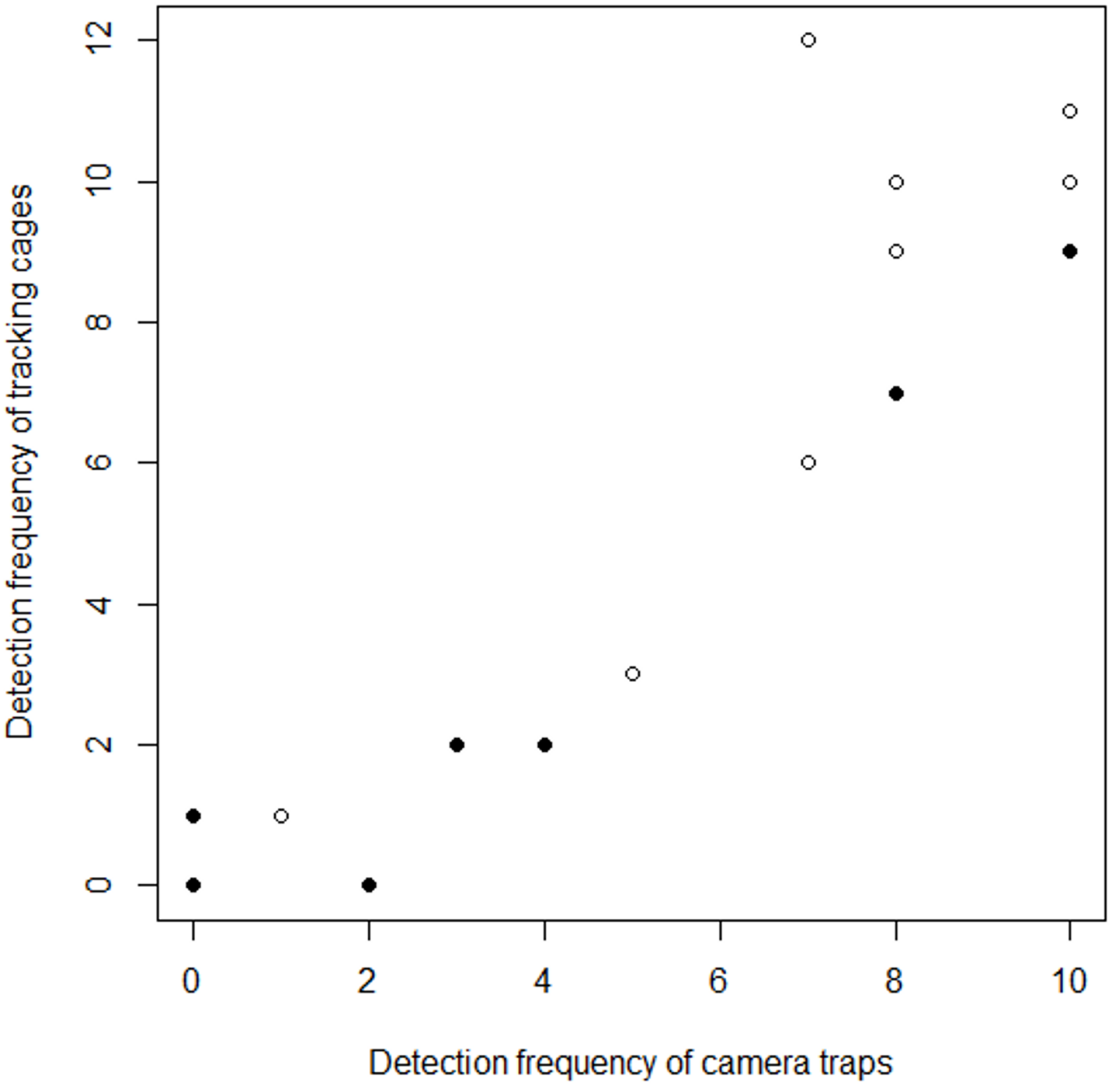
Correlation of detection rates. Correlation between paired camera trap and footprint tracking cages of the frequency of trapping sessions that detected the presence of dormice (solid circles) and wood mice (open circles) during study phases two and three combined.

There were highly significant positive correlations between camera traps and tracking cages in the frequency of sessions that paired monitoring stations detected dormice (Spearman’s rank-order correlation, *r*^*2*^ = 0.826, df = 9, *p*-value = 0.003) and wood mice (Spearman’s rank-order correlation, *r*^*2*^ = 0.833, df = 9, *p*-value = 0.003, Fig 3).

## Discussion

We have established that both camera traps and tracking cages are able to detect the presence of small, arboreal mammals at bait stations. Camera traps have rarely been used for small animals and we hope that our findings will encourage other researchers to utilise camera traps for a wider range of species, including smaller animals. As camera traps continue to become cheaper and increasingly accessible, this will become more feasible [4]. Further, we have shown that tracking stations can be adapted for use in arboreal habitats, demonstrating the advantage of continued adaptation and development of existing techniques to provide solutions for surveying elusive species.

Importantly, both methods have the potential to determine hazel dormouse presence considerably more rapidly, compared to the chief current dormouse survey techniques [27]. Our results have shown that dormice may visit bait stations and hence be detected, as soon as two days after installation. Of the stations that detected dormice, all were visited within three weeks. Whilst the methods described here require more regular visits than the recommended monthly nest box/tube checks [27], the total number of visits required is likely to be comparable, and, significantly, provide positive results much more quickly. Consequently, our methods are more flexible, such as in relation to time of year when deployed. Additionally, they can be more confidently used in a greater variety of habitats than existing survey methods, as they do not require the presence of any specific vegetation species, do not rely on dormice exhibiting nesting behaviour, and the detection probability is unlikely to be affected by the availability of natural nest sites. Lastly, as they are non-invasive, surveyors do not require a licence to use these methods.

The camera trap and footprint tracking techniques provided similar results for the majority of the trapping sessions. The estimated proportion of assumed detection failure from the two techniques was very similar, suggesting the techniques are similarly effective in detecting small mammals. The cause of failure to detect small mammal activity may be attributed to several factors, dependent on the technique in question. A qualitative comparison of the two techniques is given in S3 Fig.

### Future directions

Whilst the principle of both techniques has been proven, further work is required to establish a standardised protocol with guidelines on survey design [9]. A survey effort that minimises the risk of false absences and takes detection probability into consideration should be determined [37].

The techniques would benefit from further methodological investigation and refinement, for example, examining the effect of ecological factors such as season, abundance of dormice and bait competitors, natural food availability, habitat type and weather conditions on detection rates. Additionally, an investigation of what, if any, effect bait competitors have on dissuading focal species from visiting the monitoring stations would inform pre-baiting methodology.

In this study, the camera trap data suggest only hazel dormice and wood mice visited the bait stations. However, it is important to note it is possible that other species, such as yellow necked mice, *Apodemus flavicollis*, voles and shrews, may be present and thus leave foot prints. The characteristic metacarpal pads of the focal species, the hazel dormouse, are extremely distinctive and so are unlikely to be confused with any other small mammal species. However, wood mice prints may be confused with other rodent species [38] and so caution should be taken when distinguishing between these other small mammal species’ footprints. The adoption of statistical algorithms for footprint identification, such as those employed by Alibhai *et al*. [24] and Russell *et al*. [25] would provide a more automatic and objective method, could include a wider range of small animal species and potentially even provide additional information, such as age and sex. We envisage that the continuing development of such techniques will lead to an expansion in the use of point sampling of footprints for many taxa.

Once refined, the methods examined in this study may prove to be extremely valuable to professional ecological consultants surveying sites for dormouse presence. We envisage that they may also be beneficial to applied and academic research, such as informing habitat management planning and investigating the distribution and activity patterns of dormice and other small, arboreal mammals. Furthermore, the calibration of detection rates to accurate abundance estimates may allow the establishment of methods to determine indices of relative abundance [9, 39].

### Conclusion

Our study successfully demonstrated proof-of-concept for the use of camera traps and tracking cages to detect the presence of small, arboreal animals. As wildlife monitoring technology becomes more sophisticated and the urgent need for cheap and quick monitoring techniques heightens, it is likely that the employment of presence surveys will continue to increase. Therefore, future studies should consider these techniques when surveying for such species.

We have demonstrated that there is value in adapting and creating new survey techniques, even if established survey methods exist. Alternative techniques increase the range of potential survey methods, providing ecologists with greater flexibility to choose a technique most suitable for their particular time and financial constraints. Presence-only survey techniques need not be expensive, as exemplified by the simplicity of footprint tracking, but can dramatically reduce the delay in detection of species of conservation concern in threatened habitats.

## Supporting Information

S1 Fig Photographs of assembled footprint tracking cage. (DOCX)

S2 Fig A description of the morphological features of small mammal feet: a comparison of hazel dormice and wood mice (DOCX)

S3 Fig A comparison of the pros and cons for camera trap and tracking stations as survey techniques. (DOCX)

S1 File Survey results data table in EXCEL. (XLSX)

S2 File Camera trap video footage of dormouse and wood mouse interacting on the tracking bait cage. (AVI)
