# Supplementary material for: Take Only Photographs, Leave Only Footprints: Novel Applications of Non-Invasive Survey Methods for Rapid Detection of Small, Arboreal Animals
Source: PLoS One. 2016 Jan 20;11(1):e0146142. doi: 10.1371/journal.pone.0146142 (PMC4720397; doi:10.1371/journal.pone.0146142)

S1 Fig. Photographs of assembled footprint tracking cage: A) Side-view of tracking cage; and B) view looking down on tracking cage, with cage lid removed.


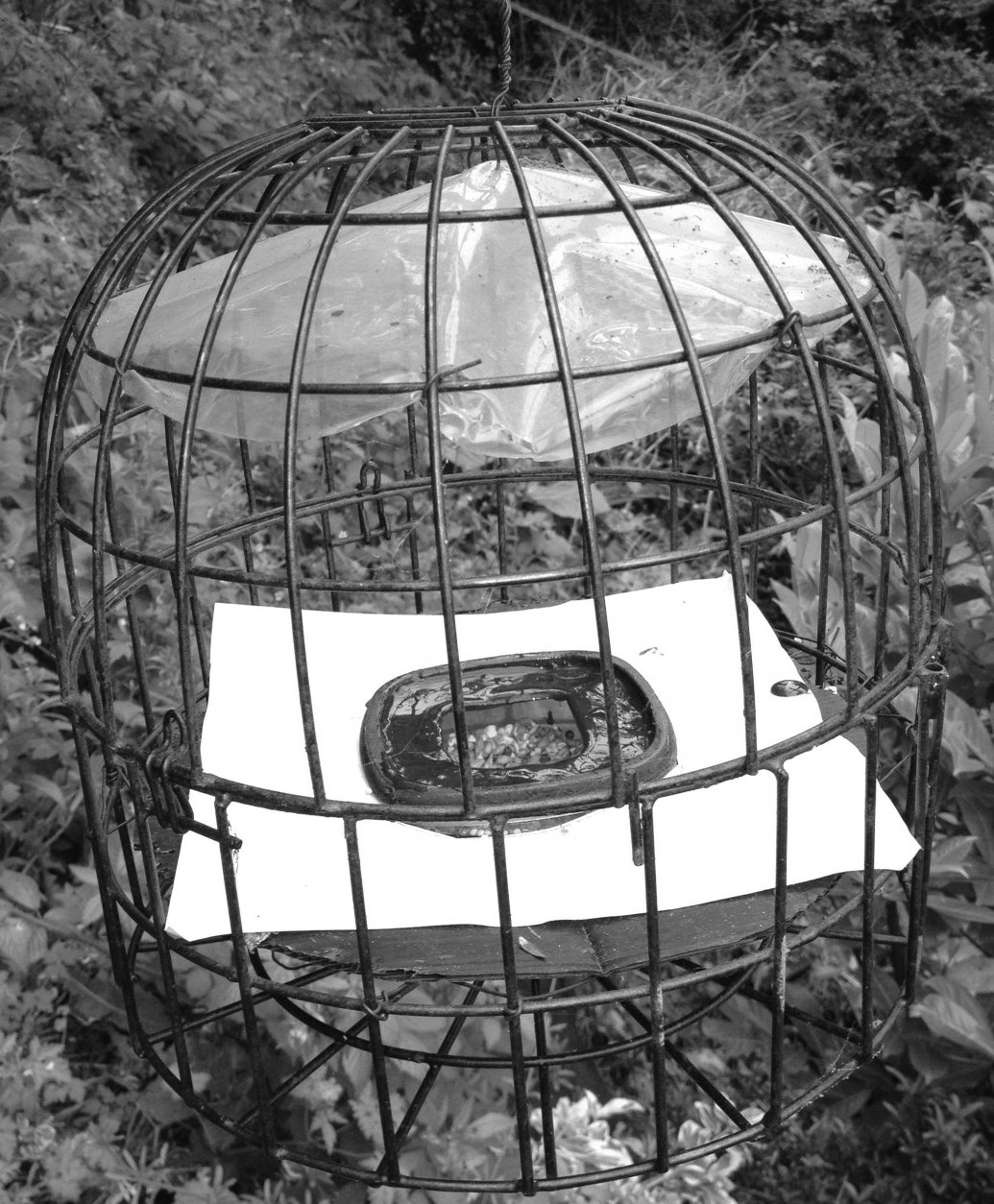


**B**

**A**


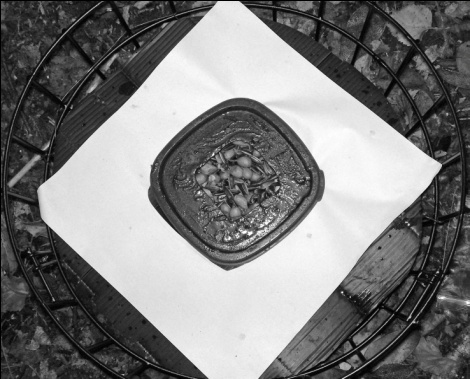

Supplement: S1 Fig — (DOCX) [file pone.0146142.s001.docx]
