# Supplementary material for: Take Only Photographs, Leave Only Footprints: Novel Applications of Non-Invasive Survey Methods for Rapid Detection of Small, Arboreal Animals
Source: PLoS One. 2016 Jan 20;11(1):e0146142. doi: 10.1371/journal.pone.0146142 (PMC4720397; doi:10.1371/journal.pone.0146142)
Supplement: S2 Fig — (DOCX) [file pone.0146142.s002.docx]

S2 Fig. A description of the morphological features of small mammal feet: a comparison of hazel dormice and wood mice from reference footprints. Main features of the feet are marked by: triangles (toes), rectangles (metacarpal pads) and circles (heel pads).

| 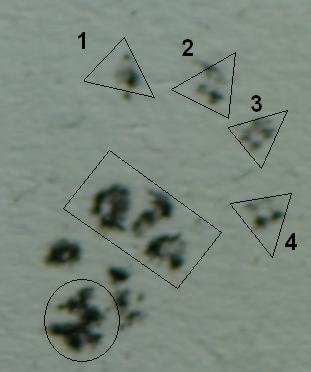 | **Dormouse fore foot**  ***Toes***   - Four toes. - Toes form a symmetrical, shallow arch. - A line drawn between toes 1 and 4 would cross well above the metacarpal pad.   ***Metacarpal pads***   - Three triangular/oval pads close together. - If excess ink may merge into an oblong.   ***Heel pads***   - Irregular concave polygon shapes. |
| --- | --- |
| 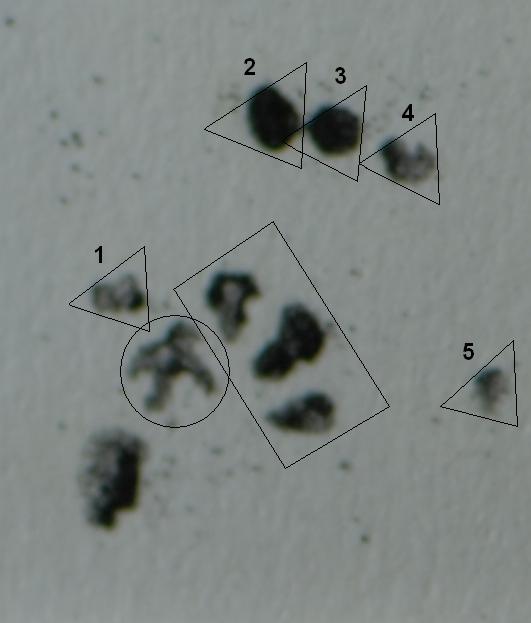 | **Dormouse hind foot**  ***Toes***   - Five toes, sometimes four as outer toe often doesn’t leave a print. - Toes 1 and 5 at an obtuse angle from middle three toes. - Angle between toes 1,2 and 5 and 1,3 and 5 generally more than 75 degrees.   ***Metacarpal pads***   - Three triangular/oval pads close together. - If excess ink may merge into an oblong.   ***Heel pads***   - Irregular concave polygon shapes. |
| 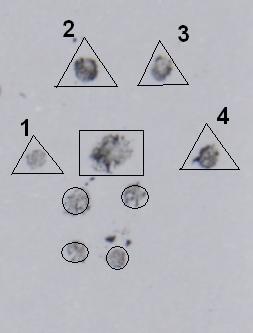 | **Wood mouse fore foot**  ***Toes***   - Four toes. - Toes form a symmetrical pattern. - A line drawn between toes 1 and 4 would transect the metacarpal pad.   ***Metacarpal pads***   - One circular pad.   ***Heel pads***   - One to four small round pads below metacarpal pads. |
| **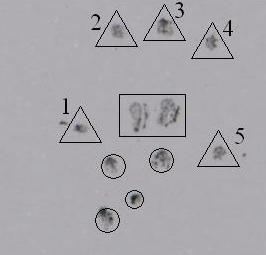** | **Wood mouse hind foot**  ***Toes***   - Five toes, sometimes four as outer toe often doesn’t leave a print. - Toes 1 and 5 at an obtuse angle from middle toe. - Angle between toes 1,2 and 5 and 1,3 and 5 generally less than 75 degrees.   ***Metacarpal pads***   - Two oval pads. - Can merge into each other if excess ink.   ***Heel pads***   - One to four small round pads below metacarpal pads |
