# Supplementary material for: Take Only Photographs, Leave Only Footprints: Novel Applications of Non-Invasive Survey Methods for Rapid Detection of Small, Arboreal Animals
Source: PLoS One. 2016 Jan 20;11(1):e0146142. doi: 10.1371/journal.pone.0146142 (PMC4720397; doi:10.1371/journal.pone.0146142)
Supplement: S3 Fig — (DOCX) [file pone.0146142.s003.docx]

S3 Fig. A comparison of the pros and cons for camera trap and tracking stations as survey techniques.

|  | **Camera trap** | **Footprint tracking** |
| --- | --- | --- |
| Invasiveness | No need to handle animals | No need to handle animals |
|  | Especially if infra-red flash is used should cause minimal disturbance (but animals may still hear/see camera trap, depending on model used [32]) | Tracking medium adheres to animal’s feet, but is non-toxic |
| Survey effort | Positive records of dormice are acquired within days to weeks | Positive records of dormice are acquired within days to weeks |
|  | Bait, batteries and memory requires replacing | Bait, tracking card and medium requires replacing |
|  | Can be left out for long periods of time before collection as stores data safely | Must collected regularly as overlapping tracks make footprint identification difficult |
|  | Higher cost reduces potential survey effort | Lower cost allows larger survey effort |
| Accuracy | Animal can be detected even if doesn’t make contact with bait | Animal must make contact with bait and tracking medium |
|  | False-negative results may result from the camera trap failing to trigger, or the delay from trigger to recording | False-negative results may result from environmental conditions damaging tracking cards |
|  | False-positives if species incorrectly identified | False-positives if species footprint incorrectly identified |
| Flexibility | Can be used in any habitat where camera traps can secured | Can be used in any habitat where tracking cages can secured |
|  | Can be used any time throughout the active dormouse season | Can be used any time throughout the active dormouse season |
| Reliability | Weather conditions may increase the number of false triggers, depleting memory and battery | Weather conditions damage tracking medium and cards, preventing tracks being left by animals |
|  | Equipment waterproof so rain and wind should not damage records | Weather conditions may destroy tracks |
|  | Potential equipment failure and user-error | Simple system reduces likelihood of equipment failure and user-error |
| Expertise | Data analysis requires minimal expertise | Data analysis requires footprint identification expertise |
|  | Placing of camera trap relative to bait requires some effort | Placing of tracking cage can be more *ad hoc* |
| Record | Permanent | Permanent |
| Costs | High initial and maintenance costs and associated security concerns | Low cost and therefore less security concerns |
